# Aging-related dysregulation in enteric dopamine and angiotensin system interactions: implications for gastrointestinal dysfunction in the elderly

**DOI:** 10.18632/oncotarget.24330

**Published:** 2018-01-29

**Authors:** Pablo Garrido-Gil, Antonio Dominguez-Meijide, Rosario Moratalla, Maria J. Guerra, Jose L. Labandeira-Garcia

**Affiliations:** ^1^ Laboratory of Neuroanatomy and Experimental Neurology, Department of Morphological Sciences, Research Center for Molecular Medicine and Chronic Diseases, University of Santiago de Compostela, Santiago de Compostela, Spain; ^2^ Networking Research Centre on Neurodegenerative Diseases, Madrid, Spain; ^3^ Instituto Cajal, Madrid, Spain

**Keywords:** aged, colon, gut, inflammation, neurotransmitters, Gerotarget

## Abstract

Gastrointestinal dysfunction is a common problem in the elderly. Aging-related changes in interactions between local dopaminergic and renin-angiotensin systems (RAS) have been observed in the brain, renal and vascular tissues. However, it is not known if these interactions also occur in the gut, and are dysregulated with aging. We showed a mutual regulation between the colonic dopaminergic system and RAS using young and aged mice deficient for major angiotensin and dopamine receptors. Aged rats showed a marked decrease in colonic dopamine D2 receptor expression, together with an increase in angiotensin type 1 (AT1) receptor expression, a decrease in angiotensin type 2 (AT2) receptor expression (i.e. an increase in the RAS pro-inflammatory arm activity), and increased levels of inflammatory and oxidative markers. Aged rats also showed increased levels of colonic dopamine and noradrenalin, and a marked decrease in acetylcholine and serotonin levels. The present observations contribute to explain an aging-related pro-inflammatory state and dysregulation in gastrointestinal function, which may be counteracted by treatment of aged animals with the AT1 receptor blocker candesartan.

## INTRODUCTION

Gastrointestinal (GI) dysfunction is a common problem in the elderly. Although different potential mechanisms may be involved, including nutritional factors or medications, a neurological disorder appears as a major factor. GI motility is controlled by several mechanisms acting both at central and local levels. Several studies have suggested neurodegeneration of the enteric nervous system as an underlying cause of GI dysregulation in the elderly, although controversy exists on which neuronal populations are lost with age [1-4], and a functional dysregulation of GI neurotransmission may be a major factor. It is known that local GI dopamine inhibits motility leading to constipation [5, 6] and acetylcholine and serotonin increase GI motility [7]. As parkinsonian patients develop early GI dysregulation and aging is the highest risk factor for Parkinson´s disease (PD), clarification of aging-related changes in GI dopaminergic system is particularly interesting.

The dopaminergic nigrostriatal system is altered during normal aging [8-10], and PD was once considered to be a form of accelerated aging [11, 12]. However, more recent studies suggest that aging does not induce a significant loss of dopaminergic neurons but rather induces changes that may increase the vulnerability of neurons to damage and increase the risk of developing PD [8, 13]. In previous studies on the nigrostriatal dopaminergic system [14], we observed that these aging-related changes include a marked decrease in the G protein-coupled dopamine type 1 (D1) and type 2 (D2) receptor expression, and a significant decrease in tyrosine hydroxylase (TH) expression and striatal dopamine levels, which is consistent with several previous findings in rodents and humans [15-18]. In addition, there exists a local renin-angiotensin system (RAS) in the nigrostriatal system. Angiotensin II (Ang II) type 1 (AT1) and type 2 (AT2) receptors are the major receptors of the RAS, and usually exert opposite actions. Aging induces an increase in the activity of the pro-oxidative pro-inflammatory RAS arm (Ang II/AT1/NADPH-oxidase/superoxide axis) and a decrease in the activity of the protective RAS arm (Ang II/AT2 axis). This is associated with the aging-related increase in levels of neuroinflammation and oxidative stress markers, and the increase in dopaminergic cell vulnerability to neurotoxins [14, 19, 20]. An important interaction between dopamine and angiotensin systems has also been observed in several studies in peripheral cells, particularly in relation to the regulation of renal and cardiovascular function [21-23], which suggests that dopamine and angiotensin systems directly regulate each other [24-26]

Both local dopaminergic (see above) and angiotensin systems have been observed in the gut. The GI RAS plays important roles in a variety of intestinal processes, including absorption, motility and inflammation [27-30], and GI RAS has also been related to GI diseases such as inflammatory bowel disease and GI motility disorders, see for review [27, 28, 30]. However, it is not known if there are functional interactions between the GI RAS and the GI dopaminergic system and if these interactions are dysregulated with aging. In the present study, colonic samples from mice deficient (ko) in different angiotensin and dopamine receptors have been used to investigate possible interactions between both systems, and colon samples from young and aged rats and mice were used to study possible aging-related changes in these interactions.

## RESULTS

### Mutual regulation of dopamine and angiotensin receptors in the proximal colon

**Figure 1:**
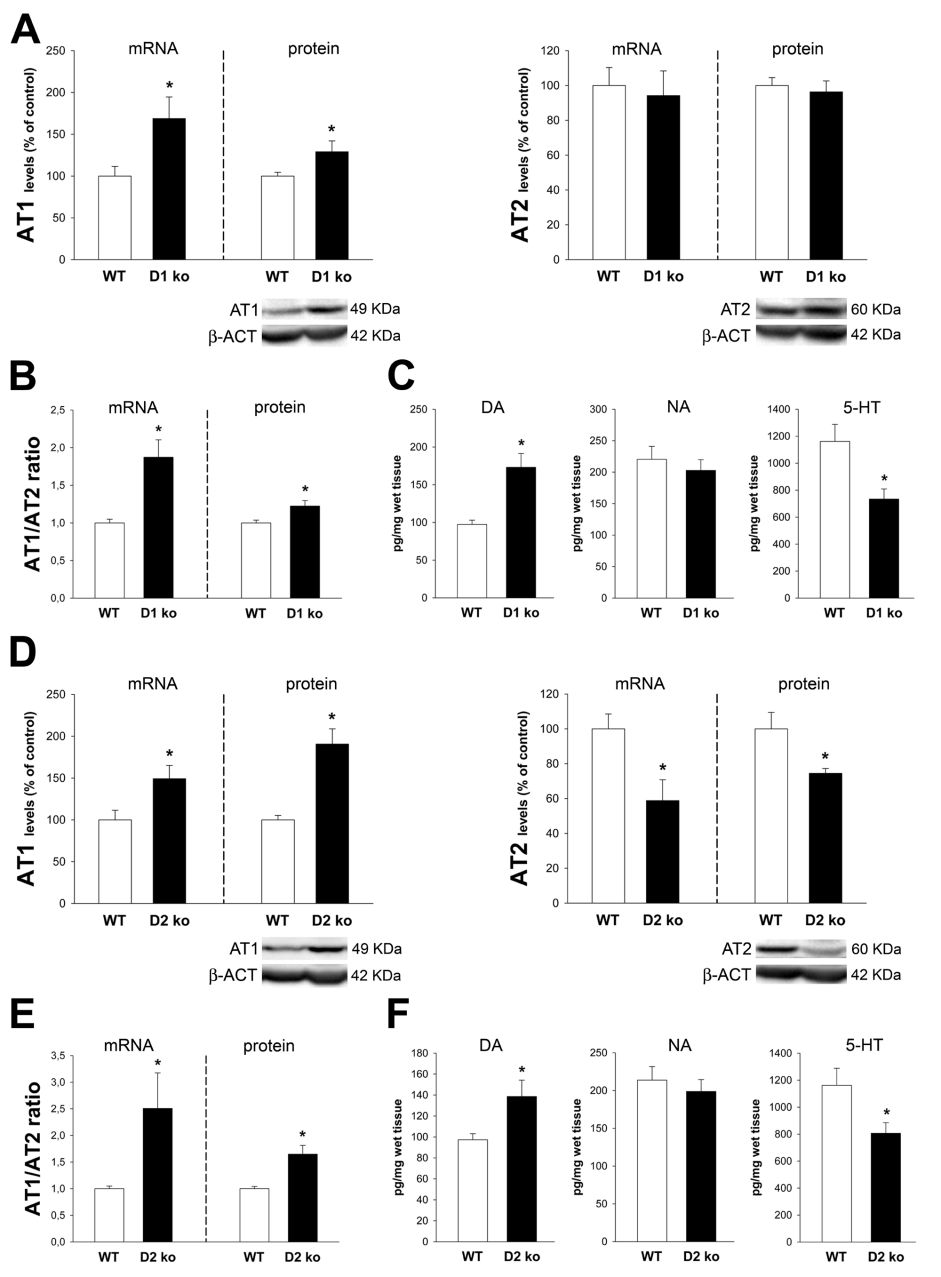
Colon of D1 receptor-deficient mice (D1-ko) and D2-deficient mice (D2-ko) Real-time quantitative RT-PCR and western blot (WB) analysis of changes in the expression of AT1 receptors, AT2 receptors and AT1/AT2 ratio, and HPLC analysis of levels of dopamine (DA), noradrenalin (NA) and serotonin (5-HT) in the ascending colon of D1-ko **A.**-**C.** and D2-ko **D.**-**F.** relative to wild-type (wt) controls. Protein expression was measured relative to the β-actin band value and the expression of each gene was measured relative to that of the housekeeping transcripts (β-Actin). PCR and WB results were normalized to the values for wild-type controls (100%). Data are means ± SEM. *p < 0.05 relative to wt controls (Student’s *t* test).

Mice deficient (ko) for D1 receptors (Figure 1A-1C) showed a significant increase in AT1 receptor expression and no significant changes in AT2 receptor expression, leading to increase in the AT1/AT2 ratio. In addition, the D1 ko mice showed increased levels of dopamine and decreased levels of serotonin with no significant changes in levels of noradrenaline. Mice ko for D2 receptors (Figure 1D-1F) showed increased expression of AT1 receptors and decreased expression of AT2 receptors, which led to a marked increase in the AT1/AT2 ratio. In addition the D2 ko mice showed increased levels of colonic dopamine and decreased levels of serotonin with no significant changes in levels of noradrenaline.

**Figure 2:**
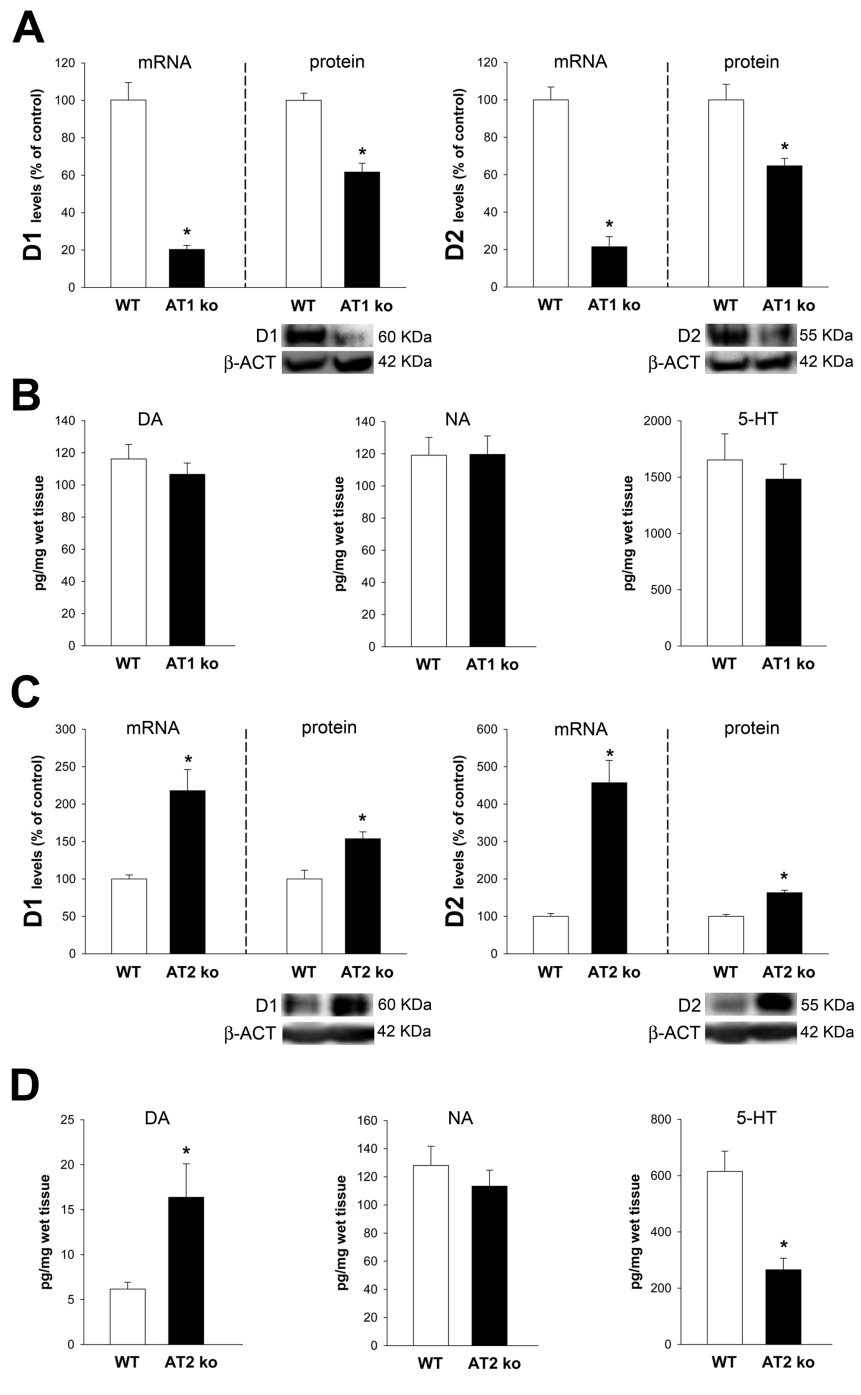
Colon of AT1 receptor-deficient mice (AT1-ko) and AT2-deficient mice (AT2-ko) Real-time quantitative RT-PCR and western blot (WB) analysis of changes in the expression of D1 receptors, D2 receptors, and HPLC analysis of levels of dopamine (DA), noradrenalin (NA) and serotonin (5-HT) in the ascending colon of AT1-ko **A.**, **B.** and AT2-ko **C.**, **D.** relative to wild-type (wt) controls. Protein expression was measured relative to the β-actin band value and the expression of each gene was measured relative to that of the housekeeping transcripts (β-actin). PCR and WB results were normalized to the values for wild-type controls (100%). Data are means ± SEM. *p < 0.05 relative to wt controls (Student’s *t* test).

Mice ko for AT1 receptors showed a marked decrease in the expression of D1 and D2 receptors (Figure 2A). However, no significant changes in levels of dopamine, serotonin or noradrenaline were observed (Figure 2B). Mice ko for AT2 receptors showed a marked increase in the expression of D1 and D2 receptors (Figure 2C). Furthermore, the proximal colon of AT2 ko mice showed increased levels of dopamine and a decrease in levels of serotonin (Figure 2D).

### The dopamine and angiotensin systems in the proximal colon of aged rats

**Figure 3:**
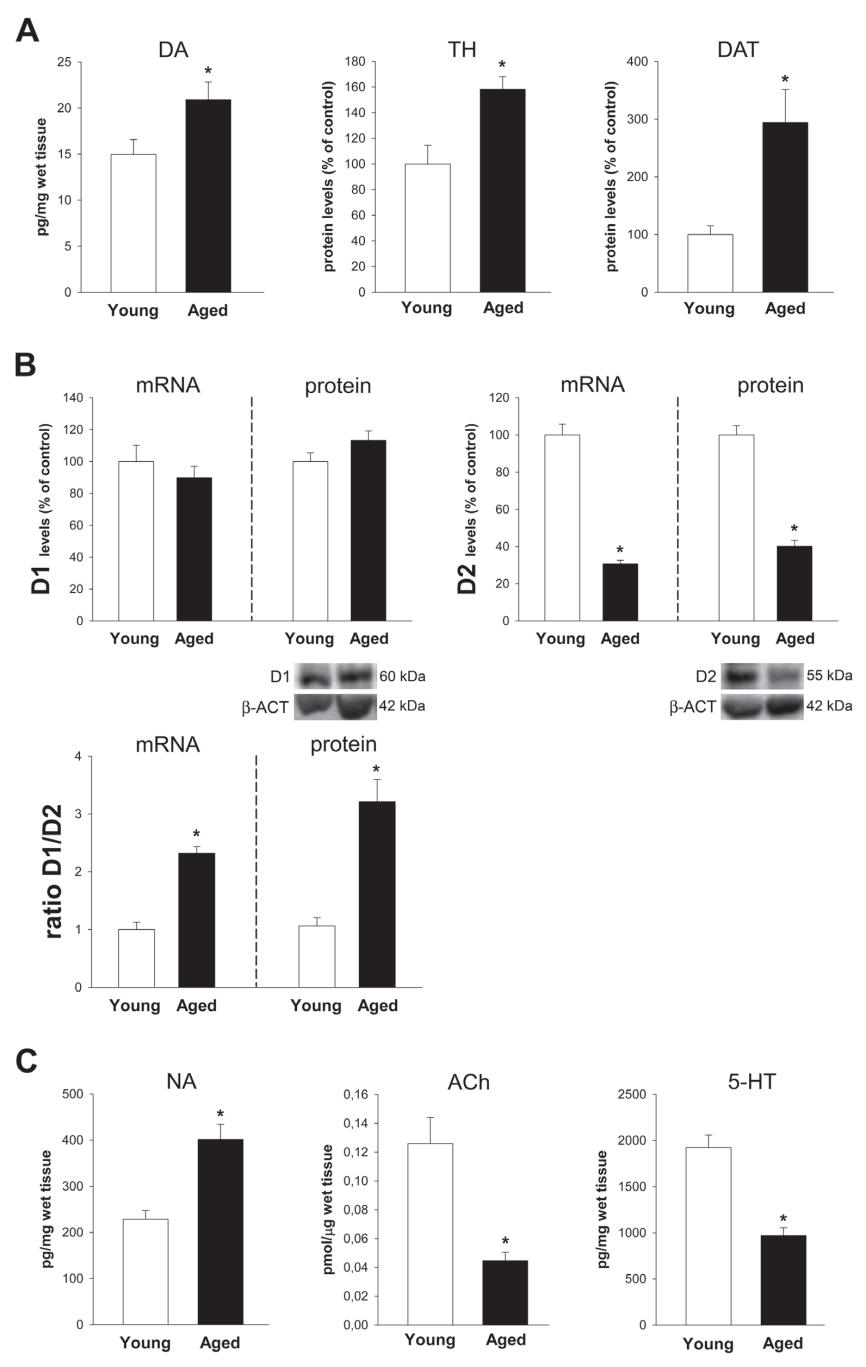
Colon of aged rats relative to young adult rats Changes in dopamine levels, and in the expression of tyrosine hydroxylase (TH), dopamine transporter (DAT) **A.**, dopamine D1 and D2 receptors and D1/D2 ratio **B.**, and levels of noradrenalin (NA), acetylcholine (Ach) and serotonin (5-HT) **C.,** in the ascending colon of aged rats relative to young adult rats. Protein expression was measured relative to the β-actin band value and the expression of each gene was measured relative to that of the housekeeping transcripts (β-actin). PCR and WB results were normalized to the values for wild-type controls (100%). Data are means ± SEM. *p < 0.05 relative to wt controls (Student’s *t* test).

Proximal colon of aged rats showed a significant increase in dopamine levels and a significant increase in the expression of TH and DAT (Figure 3A). No significant changes were observed in the expression of D1 receptors. However, a marked decrease in the expression of D2 receptors led to a marked increase in the D1/D2 ratio (Figure 3B). A significant increase in noradrenalin levels, and a marked decrease in levels of Ach and serotonin was also observed (Figure 3C).

**Figure 4:**
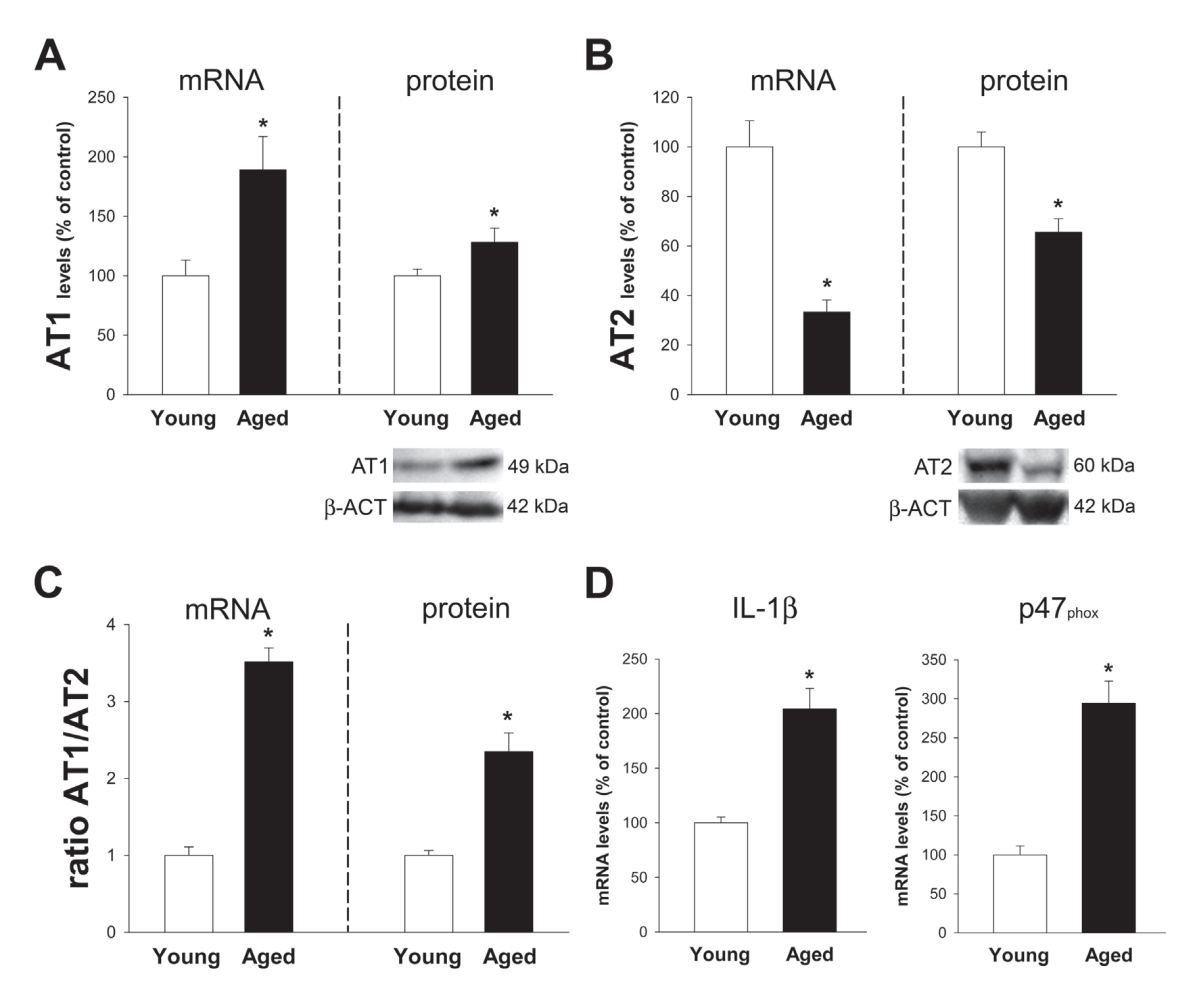
Colon of aged rats relative to young adult rats Changes in the expression of angiotensin AT1 **A.** and AT2 **B.** receptors, AT1/AT2 ratio **C.**, and levels of IL-1β and the p47^phox^ NADPH-oxidase subunit **D.** in the ascending colon of aged rats relative to young adult rats. Protein expression was measured relative to the β-actin band value and the expression of each gene was measured relative to that of the housekeeping transcripts (β-actin). PCR and WB results were normalized to the values for wild-type (WT) controls (100%). Data are means ± SEM. *p < 0.05 relative to wt controls (Student’s *t* test).

A significant upregulation of the pro-inflammatory arm of the local RAS was observed. Aged animals showed increased expression in AT1 receptors and decreased expression of AT2 receptors, both at mRNA and protein levels. This led to a marked increase in the AT1/AT2 ratio (Figure 4A-4C). Consistent with this, we observed a marked increase in the marker of NADPH-oxidase activity p47^phox^ and the marker of inflammation IL-1β (Figure 4D).

### Effect of AT1 receptor blockers and AT1 or AT2 receptor deletion

**Figure 5:**
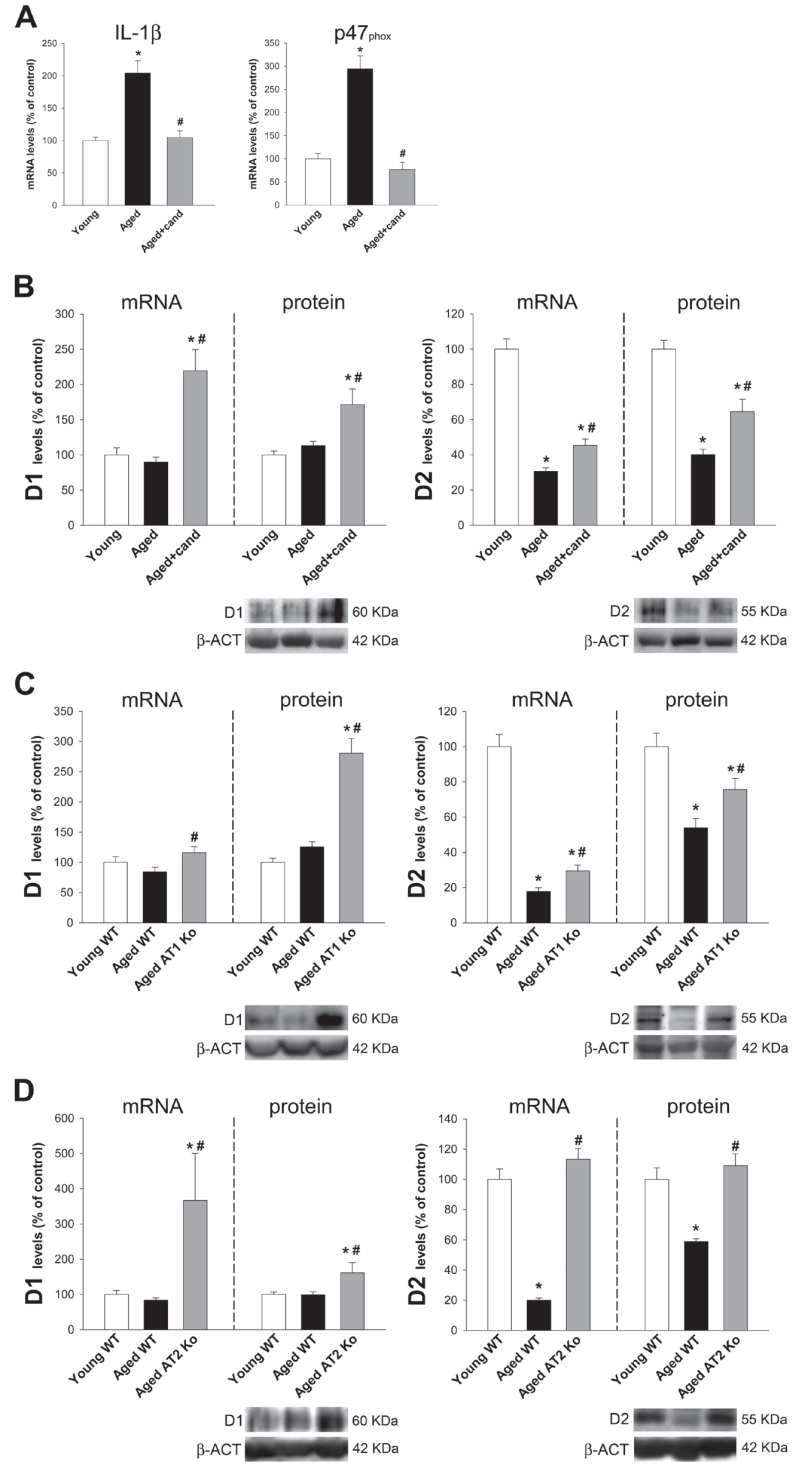
Colon of aged rats, aged rats treated with candesartan, aged AT1-ko mice and aged AT2-ko mice Changes in IL-1β and the p47^phox^ NADPH-oxidase subunit levels **A.**, and in the expression of dopamine D1 and D2 receptors in the ascending colon of aged rats, aged rats treated with candesartan **B.**, aged AT1-ko mice **C.** and aged AT2-ko mice **D.** relative to the corresponding controls (i.e. young adult rats, young wild-type, WT, mice and aged WT mice, respectively). Protein expression was measured relative to the β-actin band value and the expression of each gene was measured relative to that of the housekeeping transcripts (β-actin). PCR and WB results were normalized to the values for wild-type controls (100%). Data are means ± SEM. *p < 0.05 relative to WT controls (one-way ANOVA followed by Student-Newman-Keuls post-hoc test).

Aged rats treated with the AT1 receptor blocker candesartan showed a marked decrease in levels of the oxidative and inflammatory markers p47^phox^ and IL-1β (Figure 5A). Treatment with candesartan also induced a significant increase in the expression of D1 and D2 receptors in aged rats (Figure 5B). Consistent with this, aged AT1 deficient (ko) mice showed higher expression of D1 and D2 receptors than wild type aged mice (Figure 5C). The expression of GI dopamine D1 receptors in aged AT1 ko mice and aged rats treated with candesartan was significantly higher than in young wild type controls (no significant aging-related changes in levels of D1 receptors were observed, see above). However, D2-receptor expression in aged AT1 ko mice and aged rats treated with candesartan were significantly lower than in young controls (Figure 5B, 5C). In aged AT2 ko mice, a significant increase in D1 and D2 receptor expression was observed, as previously observed in young ko AT2 mice (Figure 5D).

## DISCUSSION

**Figure 6:**
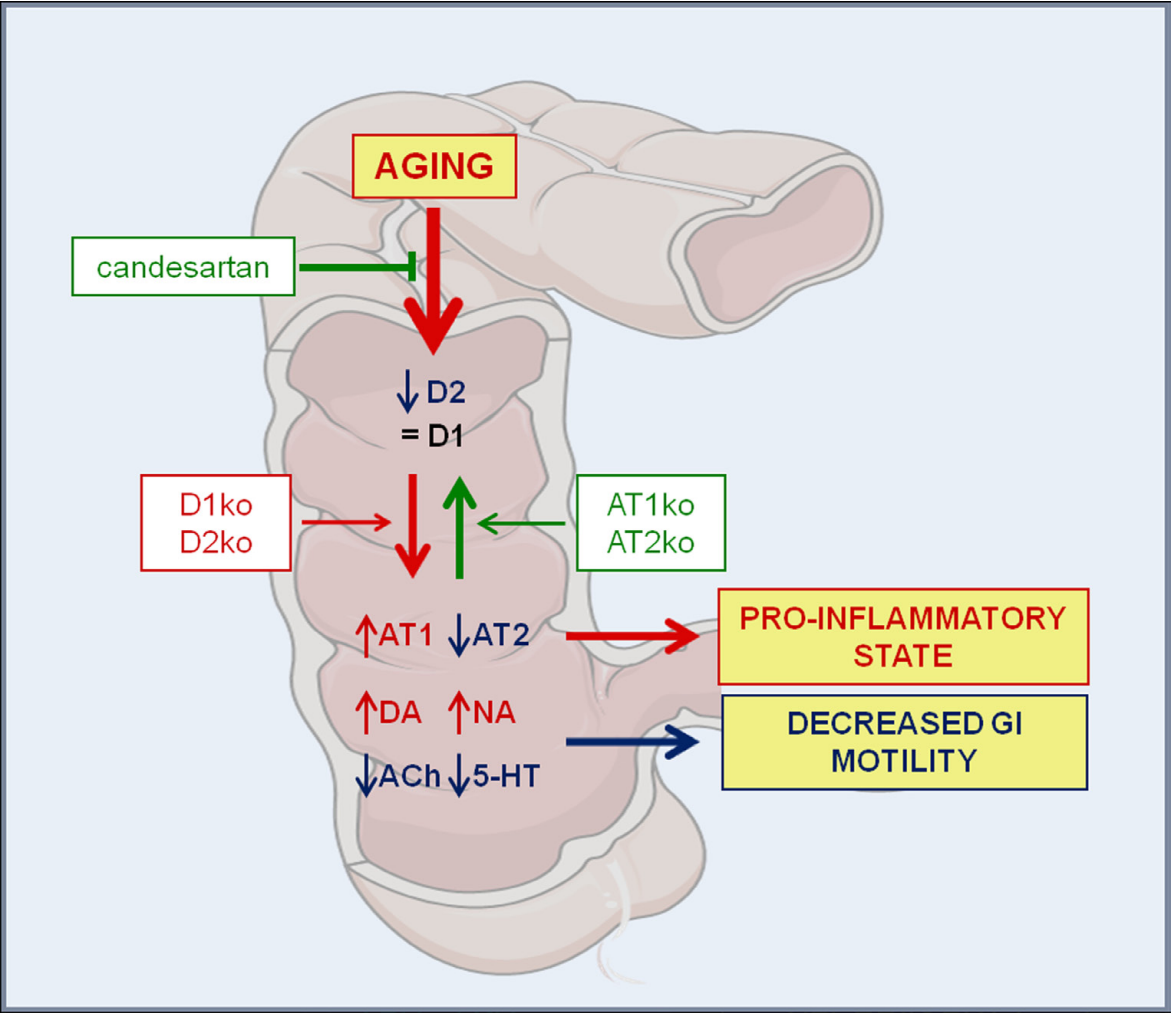
Mutual regulation between the GI dopaminergic system and GI RAS in aged rats and mice deficient for major angiotensin and dopamine receptors Aged rats showed a marked decrease in D2 receptor expression, together with an increase in AT1, a decrease in AT2 receptor expression (i.e. an increase in the RAS pro-inflammatory arm activity), an increase in levels of dopamine and noradrenalin, and a marked decrease in Ach and serotonin levels. The results contribute to explain the pro-inflammatory state and dysregulation in GI functions commonly observed in the elderly. Aging-related effects were significantly reduced by treatment with the AT1 receptor blocker candesartan. Abbreviations: 5-HT, serotonin; Ach, acetylcholine; AT1, angiotensin type 1 receptor; AT2, angiotensin type 2 receptor; DA, dopamine; D1, dopamine type 1 receptor; D2, dopamine type 2 receptor; ko, knockout; NA, noradrenalin. Figure was produced using Servier Medical Art (http://www.servier.com).

In the present experiments, we have shown a mutual regulation between the colonic dopaminergic system and colonic RAS using young and aged mice deficient for major angiotensin and dopamine receptors, and aged rats (Figure 6). In particular, mice deficient for D1 or D2 receptors showed an increase in the expression of colonic AT1 receptors and increased AT1/AT2 ratio, which may lead to increased vulnerability to GI inflammation. These mice also showed an increase in GI levels of dopamine and a decrease in serotonin levels, which may lead to decreased in GI motility. Mice deficient for AT1 receptors also showed a downregulation of D1 and D2 receptor expression. Interestingly, AT1 ko mice did not show significant changes in dopamine levels despite the decrease in D1 and D2 receptors. This suggests that AT1 receptors may mediate the increase in dopamine levels [31-33] observed in different groups of mice and rats showing upregulation of AT1 receptor expression in the present study. Opposite to that observed in AT1 ko mice, mice deficient for AT2 receptors showed upregulation of GI D1 and D2 receptor expression, increase in levels of dopamine and decrease in levels of serotonin.

The changes observed in aged rats are similar to those observed in D1 and, particularly, D2 ko mice. As observed in D2 ko mice, aged rats showed a marked increase in AT1 and decrease in AT2 expression leading to a marked increase in the AT1/AT2 ratio (i.e. the pro-inflammatory arm activity). Aged rats also showed marked decrease in Ach and serotonin levels and increased levels of noradrenalin, dopamine, TH and DAT expression. A decrease in the expression of D2 auto-receptors and an increase in TH and DAT expression may lead to increased levels of dopamine. These observations may explain the decrease in GI motility commonly observed in the elderly, as it is known that GI dopamine inhibits motility [5, 6] and Ach and serotonin increase GI motility [7].

The results observed in the different ko mice suggest that the decrease in D2 receptor expression in aged animals may play a leading role in the changes observed in angiotensin receptor expression. Aged rats also showed increased expression in AT1 and decreased expression in AT2 receptors, as observed in D2 ko mice. Therefore, it may also be possible that an initial aging-related change in GI angiotensin receptors leads to changes in dopamine receptors. However, an initial decrease in AT2 receptor activity would have led to the opposite to that observed in aged rats (i.e. increase in D2 and D1 receptors as observed in AT2 ko mice), and an initial increase in AT1 receptor expression in aged rats would have led to increased D2 expression (i.e. the opposite to that observed in ko AT1 mice) instead of the decreased D2 expression observed in aged rats.

Consistent with the increase in the activity of the RAS pro-inflammatory arm (AT1/AT2 ratio), an increase in markers of oxidative stress (p47^phox^) and inflammation (IL-1β) were observed in the colon of aged rats. This may also be a major factor for GI dysregulation in elderly. It is known that the GI RAS plays important roles in a variety of intestinal processes, including absorption and secretion, motility and inflammation [27, 28, 30]. The local GI RAS has also been related to GI diseases such as inflammatory disorders [34, 35], GI motility disorders, mesenteric ischemia and GI cancer [27-30]. The present results are also consistent with a number of studies in different tissues showing that overactivation of local or tissue RAS, via AT1 receptors and NADPH oxidase activation, mediates oxidative stress and several key events in inflammatory processes that play a major role in several aging-related diseases [36, 37]. Furthermore, the local RAS has been associated with decreased longevity and age-related degenerative changes in different tissues [38-41].

Interestingly, we observed that the aging-related increase in markers of oxidative stress and inflammation was significantly reduced by treatment of aged animals with the AT1 receptor blocker candesartan, which also induced an increase in GI D1 and D2 receptor expression. A similar effect was observed in aged AT1 ko mice. It is interesting to note that the effect of AT1 deletion in aged AT1 ko mice (i.e. increase in D1 and D2 receptor expression compared to aged WT mice) was different to that observed in young AT1 ko mice (decreased expression of D1 and D2 receptors). However, note that aged WT mice already have a marked decrease in D2 receptor expression (together with increased expression of AT1 and decreased expression of AT2). This again suggests the leading role of D2 receptor levels in dopamine/angiotensin receptor interactions.

In the nigrostriatal dopaminergic system of aged rats, we previously observed [14, 20, 42] changes in dopamine and angiotensin receptors similar to those observed in the GI system of aged animals in the present study. The substantia nigra of aged rats showed marked decrease in D1 and D2 receptor, and overactivation of the RAS proinflammatory arm (increase in AT1 and decrease in AT2 receptor expression), which was associated with higher levels of neuroinflammation and oxidative stress markers. However, we also found significant decrease in nigrostriatal TH expression and dopamine levels, which was not observed in the aged colonic samples in the present experiments. This suggests that aged central dopaminergic neurons are unable to produce normal levels of dopamine in response to the decrease in D2 auto-receptors and the increase in AT1 receptors. Both factors have been shown to increase nigrostriatal dopamine release in young animals [31-33]. However, this is not the case of GI dopaminergic neurons, since we found increased levels of dopamine in aged rats. Interestingly we (Garrido-Gil and Labandeira-Garcia, unpublished observations) and other researchers [43] have observed that a depletion in central dopamine levels (e.g. by nigral injection of the dopaminergic neurotoxin 6-hydroxydopamine) led to increased levels of GI dopamine and a decrease in GI motility. We also observed an increase in the GI RAS pro-inflammatory arm in rats with central dopaminergic depletion. Therefore, a central dopaminergic decrease in aged animals may also contribute to the observed increase in GI dopamine levels and RAS proinflammatory activity in aged rats.

In conclusion, we have shown a mutual regulation between the GI dopaminergic system and GI RAS using mice deficient for major angiotensin and dopamine receptors. Aged rats showed a marked decrease in D2 receptor expression, together with an increase in AT1, decrease in AT2 receptor expression (i.e. an increase in the RAS pro-inflammatory arm activity) and increased levels of inflammatory and oxidative markers. Aged rats also showed increased levels of dopamine and noradrenalin, and marked decrease in Ach and serotonin levels. The present observations contribute to explain the pro-inflammatory state and dysregulation in GI functions commonly observed in the elderly. Interestingly, we observed that the aging-related increase in markers of oxidative stress and inflammation was significantly reduced by treatment of aged animals with the AT1 receptor blocker candesartan, which also induced an increase in GI D1 and D2 receptor expression.

## MATERIALS AND METHODS

### Ethics statement

Investigation has been conducted in accordance with the ethical standards and according to the Declaration of Helsinki and according to national and international guidelines (European Directive 2010/63/EU and the Spanish RD/53/2013) and has been approved by the authors’ institutional review board (University of Santiago de Compostela).

Animals were housed in conditions of constant room temperature (21-22 °C) and a 12:12 hour light-dark cycle and given free access to food and water.

### Experimental design

Young adult (2 to 3-month-old) and old (18 to 20-month-old) male Sprague Dawley rats and C57/BL6 mice were included in the present study. The mice were dopamine D1 or D2 receptor knockout (D1 ko or D2 ko) mice generated by homologous recombination [44-46], and angiotensin II (Ang II) type 1 (AT1) or type 2 (AT2) receptor knockout (AT1 ko or AT2 ko) mice: AT1a ko (the major mouse AT1 isoform, and the closest murine homolog to the single human AT1; Jackson Laboratory, Bar Harbor, ME, USA) and homozygous C57BL-6 mice deficient for AT2 receptors (gift of Dr. Daniel Henrion). The corresponding wild-type (WT) littermates were used as control group. All rats and mice were divided into 5 groups. Group A comprised young adult WT mice (*n* = 12) or dopamine D1 ko (*n* = 6) or D2 ko (*n* = 6) mice, which were used to study the direct effects of dopamine receptor deficiency on colonic angiotensin AT1 and AT2 receptors and colonic neurotransmitters. Group B comprised young adult WT mice (*n* = 12) or angiotensin AT1 ko (*n* = 6) or AT2 ko (*n* = 6) mice, which were used to study the effects of deficiency of angiotensin receptors on colonic dopamine D1 and D2 receptors and colonic neurotransmitters. Group C was composed of young adult (*n* = 6) and old (*n* = 6) male rats. This group was used to study the effects of aging on the colonic dopaminergic system (D1 and D2 receptor expression and levels of dopamine, tyrosine hydroxylase, TH, or dopamine transporter, DAT), the colonic RAS (AT1 and AT2 receptor expression and levels of the NADPH-oxidase subunit p47^phox^ and the cytokine interleukin-1β; IL-1β) and other colonic neurotransmitters (noradrenalin, serotonin or acetylcholine). Group D comprised young adult (*n* = 6) and old (*n* = 12) male rats which were treated with the angiotensin AT1 receptor antagonist candesartan cilexetil (*n* = 6) (1 mg/kg/day; Astra Zeneca; orally in “Nocilla” hazelnut-cream spread; Nutrexpa, Barcelona, Spain) or vehicle (*n* = 6) for 2 weeks. This group was used to study the effects of AT1 receptor blockade on aging-related dysregulation of the RAS proinflammatory arm (levels of p47^phox^ and IL-1β) and dopamine system (D1 and D2 receptor expression). In order to confirm the effects of the pharmacological blockade of AT1 receptor, we included an additional group E of animals which was composed of young WT (*n* = 12) and old WT (*n* = 12) or old AT1 ko (*n* = 6) or old AT2 ko (*n* = 6) mice. Mice and rats were killed by decapitation and the proximal (ascending) colon was rapidly removed, carefully cleaned with cold isotonic phosphate buffer saline and fragmented in longitudinal sections (5 mm-length) on an ice-cold plate. Finally, the proximal colon sections were frozen on dry ice and stored at -80 °C until analysis.

### Western blot analysis

Protein levels of TH, DAT, dopamine D1 and D2 receptors and angiotensin AT1 and AT2 receptors were analyzed by western blot (WB). Briefly, colonic tissue was homogenized in RIPA buffer containing protease inhibitor cocktail and PMSF and centrifuged at 14000 rpm for 20 min at 4°C. Then, the Pierce BCA protein assay was used to determine total protein concentration. Subsequently, equal amounts of protein were separated by 5%-10% bis-tris polyacrylamide gel, and transferred to nitrocellulose membrane. The membranes were incubated overnight with primary antibodies against TH (AB152, Millipore; 1:5000), D1 (SAB4500671, Sigma; 1:800) or the following antibodies from Santa Cruz Biotechnology (diluted 1:200): D2 (sc-9113), DAT (sc-1433), AT1 (sc-31181) and AT2 (sc-9040). Then, the membranes were incubated 1h at room temperature with the corresponding horseradish peroxidase (HRP) conjugated secondary antibody from Santa Cruz Biotechnology (1:2500): chicken anti mouse (sc-2954), goat anti rabbit (sc-2004) and donkey anti goat (sc-2020). Immunoreactivity was detected with the Luminata Crescendo Western HRP Substrate (Millipore) and visualized with a chemiluminescence detection system (Molecular Imager ChemiDoc XRS System, BioRad). Blots were stripped and reincubated with an antibody against β-actin (A2228, Sigma; 1:10000) as loading control. In each animal, protein expression was measured by densitometry of the corresponding band and expressed relative to the β-actin band value. The data were then normalized to the values of the control group of the same batch (100%) to counteract any between- batch variability. Finally, the results were expressed as mean ± SEM.

### RNA extraction and real-time quantitative PCR

RT-PCR analysis was used to measure the messenger RNA (mRNA) levels of dopamine D1 and D2 receptors, angiotensin AT1 and AT2 receptors, IL-1β and p47^phox^. Firstly, total RNA from the colon region was extracted with Trizol (Invitrogen) according to the manufacturer’s instructions. Then, total RNA concentration was estimated using a Nanodrop (Thermo Scientific) and 2.5 µg was reverse transcribed to complementary DNA with deoxynucleotide triphosphate, random primers, and the Moloney murine leukemia virus reverse transcriptase (200 U; Invitrogen). Finally, the real-time PCR analysis was performed with a real-time iCycler PCR platform (Bio-Rad) and IQ SYBR Green Supermix kit (BioRad). β-actin was used as housekeeping gene and was amplified in parallel with the genes of interest. The relative levels of mRNA were evaluated by the delta Ct method (2-ΔΔCt), where Ct is the cycle threshold. Expression of each gene was obtained as relative to the housekeeping transcripts. Forward (F) and reverse (R) primers were designed for each gene by using Beacon Designer software (Premier Biosoft).

Primers sequences were as follows: For β-actin, forward (F) 5’-TCGTGCGTGACATTAAAGAG-3’, reverse (R) 5’-TGCCACAGGATTCCATACC-3’; for mouse AT1a receptor, F 5’-GCTAACCTGGAGTCATCAAG-3’, R 5’-ACTAACTGGCATTGTTTGGG-3’; for mouse AT2, F 5’-TGTAATCAGCCTAGCCATTG-3’, R 5’-CTACTTGACTTCCTGTTCTCG-3’; for mouse D1 receptor, F 5’-CAGCGTGGACAGGTATTGG-3’, R 5’-TGGAATGAAGGATATGAGAACAGA-3’; for mouse D2 receptor, F 5’-GCAGACCACCACCAACTA-3’, R 5’-CCACCACCTCCAGATAGAC-3’; for rat AT1a receptor, F 5’-TTCAACCTCTACGCCAGTGTG-3’, R 5’-GCCAAGCCAGCCATCAGC-3’; for rat AT2, F 5’-AACATCTGCTGAAGACCAATAG -3’, R 5’-AGAAGGTCAGAACATGGAAGG- 3’; for rat D1 receptor, F 5’-CGGGCTGCCAGCGGAGAG 3’, R 5’-TGCCCAGGAGAGTGGACAGG-3’; for rat D2 receptor, F 5’- AGACGATGAGCCGCAGAAAG-3’, R 5’- GCAGCCAGCAGATGATGAAC-3’; for rat IL-1 β, F 5-AGGACCCAAGCACCTTCTTT-3’, R 5-AGACAGCACGAGGCATTTTT-3’; and for rat p47^phox^, F 5-CCACACCTCTTGAACTTCTTC-3’, R 5-CTCGTAGTCAGCGATGGC-3’

### High performance liquid chromatography

Measurements of dopamine, noradrenalin and serotonin levels were performed by HPLC analysis, as previously described [31]. Briefly, colonic tissue was homogenized and then centrifuged at 4°C (14000g; 10min). The remaining solution was filtered and injected (20 µl/injection) into the HPLC system (Shimadzu LC prominence). Separation of each neurotransmitter was performed with the aid of a reverse phase analytical column (Waters Symmetry 300C18). The mobile phase consisted of a 10% MeOH solution (pH 4) containing 70 mM KH2PO4, 1 mM octanesulfonic acid and 1 mM and was delivered at a rate of 1 ml/min. A coulometric electrochemical detector (ESA Coulochem III) was used for signal detection. The first and the second electrode of the analytical cell were set at +50 mV and +350 mV, respectively, and the guard cell was set at -100 mV. Data were processed with the Shimadzu LC solution software and were expressed as picogram per milligram of wet tissue.

### Acetylcholine analysis

Acetylcholine (Ach) was measured in the proximal colon by using the choline/acetylcholine assay kit (Abcam, Cambridge) following the manufacturer’s instructions. Data was expressed as pmol per µg of wet tissue.

### Statistical analysis

All data were obtained from at least three independent experiments and were expressed as means ± SEM. Two group comparisons were analyzed by a *Student’s t* test, and multiple comparisons were analyzed by one-way ANOVA followed by Student-Newman-Keuls post-hoc test. Differences were considered statistically significant at *p* ≤0.05. Statistical analyses were carried out with SigmaStat 3.0 from Jandel Scientific (San Rafael, CA, USA).

## Abbreviations

Ach: acetylcholine
Ang II: angiotensin II
AT1: angiotensin type 1
AT2: angiotensin type 2
DAT: dopamine transporter
GI: gastrointestinal
HPLC: high performance liquid chromatography
HRP: horseradish peroxidase
IL-1β: interleukin-1β
Ko: knockout
PD: Parkinson´s disease
RAS: renin-angiotensin systems
TH: tyrosine hydroxylase
WB: western blot
WT: wild-type
